# *In Vitro* and *In Vivo* Efficacy of a Novel and Long-Acting Fungicidal Azole, PC1244, on Aspergillus fumigatus Infection

**DOI:** 10.1128/AAC.01941-17

**Published:** 2018-04-26

**Authors:** Thomas Colley, Gurpreet Sehra, Anuradha Chowdhary, Alexandre Alanio, Steven L. Kelly, Yasuo Kizawa, Darius Armstrong-James, Matthew C. Fisher, Andrew G. S. Warrilow, Josie E. Parker, Diane E. Kelly, Genki Kimura, Yuki Nishimoto, Mihiro Sunose, Stuart Onions, Damien Crepin, Franz Lagasse, Matthew Crittall, Jonathan Shannon, Matthew McConville, John King-Underwood, Alan Naylor, Stéphane Bretagne, John Murray, Kazuhiro Ito, Pete Strong, Garth Rapeport

**Affiliations:** a Pulmocide Ltd., London, United Kingdom; b Department of Medical Mycology, Vallabhbhai Patel Chest Institute, University of Delhi, Delhi, India; c Institut Pasteur, CNRS, Molecular Mycology Unit, French National Reference Center for Invasive Mycoses & Antifungals, URA3012, Paris, France; d Paris Diderot, Sorbonne Paris Cité University, Paris, France; e Parasitology-Mycology Laboratory, Saint Louis Hospital, Assistance Publique-Hôpitaux de Paris (APHP), Paris, France; f Centre for Cytochrome P450 Biodiversity, Institute of Life Science, Swansea University Medical School, Swansea, Wales, United Kingdom; g Laboratory of Physiology and Anatomy, School of Pharmacy, Nihon University, Funabashi, Japan; h Department of Microbiology, Royal Brompton Hospital, London, United Kingdom; i National Heart and Lung Institute, Imperial College School of Medicine, London, United Kingdom; j Department of Infectious Disease Epidemiology, Imperial College School of Public Health, St. Mary's Campus, London, United Kingdom; k Sygnature Discovery Ltd., Nottingham, United Kingdom; l CompChem Resource, Ledbury, United Kingdom; m Alan Naylor Consultancy Ltd., Royston, United Kingdom

**Keywords:** Aspergillus fumigatus, azole, inhalation, CYP51, azole resistant, triazole

## Abstract

The antifungal effects of the novel triazole PC1244, designed for topical or inhaled administration, against Aspergillus fumigatus were tested in a range of *in vitro* and *in vivo* studies. PC1244 demonstrated potent antifungal activities against clinical A. fumigatus isolates (*n* = 96) with a MIC range of 0.016 to 0.25 μg/ml, whereas the MIC range for voriconazole was 0.25 to 0.5 μg/ml. PC1244 was a strong tight-binding inhibitor of recombinant A. fumigatus CYP51A and CYP51B (sterol 14α-demethylase) enzymes and strongly inhibited ergosterol synthesis in A. fumigatus with a 50% inhibitory concentration of 8 nM. PC1244 was effective against a broad spectrum of pathogenic fungi (MIC range, <0.0078 to 2 μg/ml), especially Aspergillus terreus, Trichophyton rubrum, Candida albicans, Candida glabrata, Candida krusei, Cryptococcus gattii, Cryptococcus neoformans, and Rhizopus oryzae. PC1244 also proved to be quickly absorbed into both A. fumigatus hyphae and bronchial epithelial cells, producing persistent antifungal effects. In addition, PC1244 showed fungicidal activity (minimum fungicidal concentration, 2 μg/ml) which indicated that it was 8-fold more potent than voriconazole. *In vivo*, once-daily intranasal administration of PC1244 (3.2 to 80 μg/ml) to temporarily neutropenic, immunocompromised mice 24 h after inoculation with itraconazole-susceptible A. fumigatus substantially reduced the fungal load in the lung, the galactomannan concentration in serum, and circulating inflammatory cytokine levels. Furthermore, 7 days of extended prophylaxis with PC1244 showed *in vivo* effects superior to those of 1 day of prophylactic treatment, suggesting accumulation of the effects of PC1244. Thus, PC1244 has the potential to be a novel therapy for the treatment of A. fumigatus infection in the lungs of humans.

## INTRODUCTION

The incidence of fungal infections has increased substantially over the past 2 decades, and invasive forms are leading causes of morbidity and mortality, especially among immunocompromised or immunosuppressed patients. In addition, chronic lung infections with Aspergillus in patients with, for example, a previous infection with Mycobacterium tuberculosis (1) or pulmonary inflammatory diseases can leave patients with poor lung function and extensive and permanent lung structural changes (2–4).

Systemic triazole therapy is the basis for treating infections with pathogenic fungi, but the adverse effects of itraconazole (ITC), voriconazole (VRC), and posaconazole (POS) are well characterized and thought to be a consequence of the pharmacological effects of the compounds in host tissues (5–9). It has been observed that up to 15% of patients treated with VRC experience raised transaminase levels in the liver, a site of triazole toxicity (10, 11). Serious unwanted effects in other organs have been reported after oral or systemic VRC and POS treatment, and exposure of the liver also results in significant drug interactions arising from triazole inhibition of hepatic cytochrome P450 (CYP) enzymes (12, 13), although the recent azoles isavuconazole and VT1161 showed better risk-benefit profiles in clinical or preclinical tests (14, 15).

Administration of triazoles orally can lead to wide variations in patient response due to variable plasma concentrations, leading to compromised individual efficacy (16). Furthermore, notable drug interactions for VRC due to the inhibition of hepatic cytochrome P450 enzymes make its clinical use challenging, and indeed, the variability in exposure of the triazoles when they are administered via the oral route necessitates the need for close therapeutic drug monitoring and limits the use of triazole therapy prophylactically in at-risk groups (13, 16). In addition, structural changes in the lung architecture, caused by chronic pulmonary disease or infection with M. tuberculosis, can lead to Aspergillus colonization of preexisting cavities, limiting the efficacy of orally administered compounds, which often struggle to penetrate into the pulmonary epithelial lining fluid (17). It is acknowledged that targeted administration to the lung, the primary point of infection, would prolong the lung tissue residence time and reduce systemic exposure to display a better risk-benefit ratio. Recently, existing antifungal medications, such as amphotericin B, VRC, and ITC, have been repurposed in this manner to effectively prevent invasive disease (18–20).

**FIG 1:**
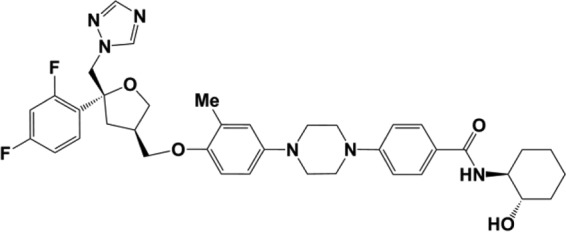
Structure of PC1244. Me, methyl.

In this report, we disclose the *in vitro* and *in vivo* activities of an antifungal agent of the newly discovered azole class, 4-(4-(4-(((3*R*,5*R*)-5-((1*H*-1,2,4-triazol-1-yl)methyl)-5-(2,4-difluorophenyl)tetrahydrofuran-3-yl)methoxy)-3-methylphenyl)piperazin-1-yl)-*N*-((1*S*,2*S*)-2-hydroxycyclohexyl)benzamide (referred to here as PC1244; Fig. 1) (21). The compound demonstrates activities comparable to those of POS and superior to those of VRC against both ITC-susceptible and -resistant strains, and it has been designed to have physicochemical properties suitable for topical administration to the lung and promote long-lasting tissue residency.

## RESULTS

### *In vitro* antifungal activity against laboratory-adapted strains of Aspergillus fumigatus.

 The antifungal activities of the test compounds against A. fumigatus strains (itraconazole [ITC]-susceptible strains NCPF2010, AF294, and AF293; ITC-resistant strains AF72 and AF91) were calculated from growth curves generated by spectrophotometric analysis and compared to the activities against positive and negative controls.

It was observed that the concentrations of PC1244 needed to achieve the endpoints of 50% inhibition (50% inhibitory concentration [IC_50_]) and 90% inhibition (IC_90_) (IC_50_s and IC_90_s were determined from optical density [OD] measurements) were significantly lower than those of all reference compounds, including voriconazole (VRC), posaconazole (POS), and itraconazole (ITC), against ITC-susceptible A. fumigatus laboratory strains (NCPF2010, AF294, AF293) (Table 1) (22, 23). In addition, PC1244 was the most active test agent against known ITC-resistant A. fumigatus strains (AF72, AF91) (24, 25) (Table 1).

**TABLE 1:** Antifungal effects of PC1244 and known antifungal agents against azole-susceptible and azole-resistant strains of A. fumigatus^*a*^

Strain	IC_50_ (IC_90_) (μg/ml) of the indicated agent					
	PC1244	Voriconazole	Posaconazole	Itraconazole	Amphotericin B	Caspofungin
NCPF2010	0.0017 (0.0022)	0.15 (0.21)	0.0070 (0.0084)	0.037 (0.054)	0.20 (0.62)	0.065 (>1)
AF294	0.0021 (0.0041)	0.083 (0.27)	0.0056 (0.011)	0.041 (0.052)	0.21 (0.79)	>1 (>1)
AF293	0.0026 (0.012)	0.25 (0.74)	0.010 (0.028)	0.032 (0.23)	0.24 (0.85)	>1 (>1)
AF72	0.0024 (0.026)	0.025 (0.066)	0.042 (0.30)	0.31 (>1)	0.12 (0.42)	0.065 (>1)
AF91	0.0037 (0.024)	0.14 (0.28)	0.038 (0.049)	0.22 (>1)	0.28 (0.75)	0.11 (>1)

a IC_50_ and IC_90_ values were determined from optical density measurements. All compounds were tested over a range of concentrations (0.002 to 1 μg/ml). The data are from three independent experiments, and each test was performed in quadruplicate.

### *In vitro* antifungal activity against clinically isolated A. fumigatus strains.

**FIG 2:**
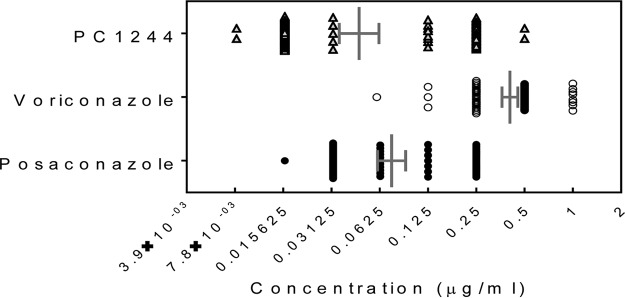
Inhibitory activity of PC1244 against 96 clinical isolates of A. fumigatus from France and the United Kingdom. Each horizontal bar presents the geometric mean with the 95% confidence interval.

The antifungal activity of PC1244 was further evaluated against 96 clinical isolates (obtained from Saint Louis Hospital, Paris, France [50 isolates], and the North West England Mycology Reference Centre, Manchester, UK [46 isolates]). In this study, PC1244 was found to be 6.2-fold more potent than VRC and demonstrated effects comparable to those of POS on the basis of their geometric mean MICs (Table 2; Fig. 2). During this assay, the quality control strain A. fumigatus ATCC 204305 was used for validation, and posaconazole showed a MIC of 0.25 μg/ml, which was within the range set by EUCAST guidelines.

**TABLE 2:** *In vitro* activities of PC1244, posaconazole, and voriconazole against 96 clinically isolated A. fumigatus strains

Test agent	MIC (μg/ml)^*a*^				
	Range	Geometric mean	Mode	50%	90%
PC1244	0.008–2	0.067^*b*^	0.016	0.032	0.50
Voriconazole	0.06–4	0.42	0.50	0.50	1.0
Posaconazole	0.016–2	0.10^*b*^	0.032	0.063	0.50

a All MICs were determined visually.

b *P* < 0.0001 versus the results for voriconazole (one-way ANOVA with Tukey's test).

### *In vitro* assessment of antifungal activity using the CLSI methodology.

A. fumigatus growth inhibition by PC1244 was confirmed by the Clinical and Laboratory Standards Institute (CLSI) method as well as the EUCAST microdilution method. Following the CLSI methodology guidelines (document M38-A [26]), the growth of four ITC-susceptible laboratory A. fumigatus strains was assessed visually. PC1244 generated a MIC (0.063 μg/ml) which was comparable to that of POS (MIC, 0.047 μg/ml) and which indicated that it was 8-fold more potent than VRC (MIC, 0.5 μg/ml).

### CYP51 binding properties.

**FIG 3:**
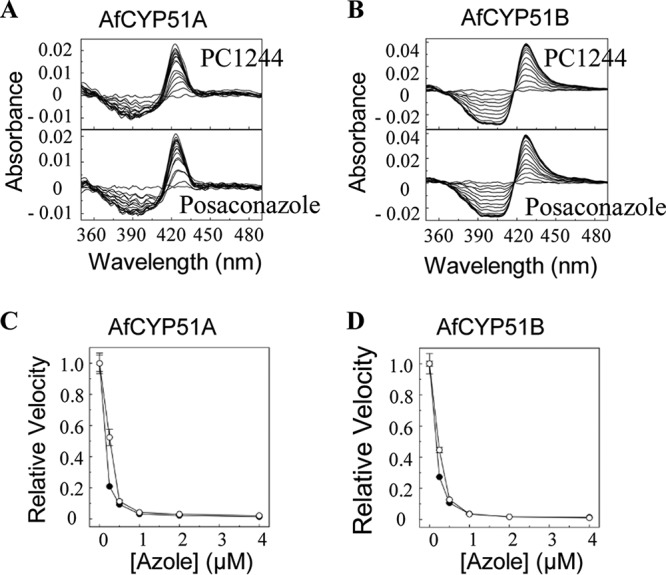
Efficacy of PC1244 on sterol 14α-demethylase (CYP51) activity. (A, B) Type II azole binding spectra for A. fumigatus CYP51A (AfCYP51A) (A) and CYP51B (AfCYP51B) (B). Each experiment was performed 4 to 6 times, although only one replicate is shown, (C, D) IC_50_s of posaconazole (●) and PC1244 (○). Mean relative velocity values with standard deviations are shown for A. fumigatus CYP51A (C) and CYP51B (D).

Both PC1244 and POS produced type II difference spectra when titrated against purified recombinant A. fumigatus (AF293) CYP51A and CYP51B enzymes. PC1244 bound to the two isoenzymes with affinities similar to those of POS (for CYP51A, dissociation constant [*K_d_*] values were 0.74 and 0.96 μM for PC1244 and POS, respectively; for CYP51B, *K_d_* values were 0.018 and 0.012 μM, respectively) (Fig. 3A and B). The low-end accuracy limit for *K_d_* determinations using the modified Morrison equation is ∼0.5 to 1% of the enzyme concentration (27), i.e., 0.020 to 0.040 μM in this study. Consequently, calculated *K_d_* values below 0.020 μM should be treated numerically as <0.020 μM.

### Inhibitory activity against A. fumigatus CYP51 enzymes.

The inhibitory activities of PC1244 against A. fumigatus sterol 14α-demethylases were determined using 0.5 μM A. fumigatus CYP51A and 0.5 μM CYP51B in the membrane fraction prepared from Escherichia coli expression clones and compared to those of posaconazole. Both PC1244 and POS were strong tight-binding inhibitors of CYP51A and CYP51B *in vitro* (CYP51A IC_50_s for PC1244 and POS, 0.27 and 0.16 μM, respectively; CYP51B IC_50_s for PC1244 and POS, 0.23 and 0.17 μM, respectively), suggesting apparent *K_i_* values below 1 nM (27), with PC1244 being equally as effective as POS (Fig. 3C and D). These data suggest that both agents share the same mode of action by directly coordinating as the sixth axial ligand of the CYP51 heme iron.

### Cell-based A. fumigatus sterol composition and CYP51 assay.

The sterol composition of A. fumigatus (NCPF2010) was determined for cells treated with 0, 0.0001, 0.001, 0.01, 0.1, and 1 μg/ml PC1244 and posaconazole. Sterols were extracted by saponification with KOH followed by extraction with *n*-hexane and trimethylsilyl derivatization prior to analysis by gas chromatography-mass spectrometry (GC-MS). Azole treatment of A. fumigatus resulted in the dose-dependent accumulation of the 14α-methylated sterols (lanosterol and eburicol) and the corresponding depletion of the final sterol product, ergosterol (Table 3), characteristic of cellular CYP51 inhibition.

**TABLE 3:** Sterol composition of A. fumigatus isolates treated with either posaconazole or PC1244

Treatment and sterol	Sterol composition^*a*^ (%) after treatment with DMSO or the indicated compound at the following concn (μg/ml):					
	DMSO	0.0001	0.001	0.01	0.1	1
Posaconazole						
Ergosterol	100 ± 0	94.5 ± 0.1	87.2 ± 0.7	74.7 ± 0.8	67.8 ± 0.3	67.4 ± 1.5
Ergost-5,7-dienol	0	3.3 ± 1.9	3.9 ± 0.6	0	0	0
Lanosterol	0	0	3.0 ± 0.9	7.0 ± 0.3	8.8 ± 0.5	8.8 ± 0.4
Eburicol	0	2.2 ± 1.2	5.9 ± 0.5	18.3 ± 1.1	23.4 ± 0.8	23.8 ± 1.3
PC1244						
Ergosterol	100 ± 0	91.3 ± 0.1	89.2 ± 0.2	76.8 ± 2.6	61.0 ± 1.6	58.7 ± 2.2
Ergost-5,7-dienol	0	4.6 ± 1.8	4.6 ± 0.8	0	0	0
Lanosterol	0	1.7 ± 0.7	2.8 ± 0.6	8.5 ± 1.1	12.3 ± 0.8	13.1 ± 0.9
Eburicol	0	2.5 ± 1.1	3.4 ± 1.2	14.7 ± 1.5	26.7 ± 0.8	28.2 ± 1.3

a The values are the means from three biological replicates ± standard deviations of the means.

CYP51 enzyme-inhibitory activity was also measured in a cell-based assay, as described previously (28). In this plate-based ergosterol quantification experiment, oxidation of ergosterol by cholesterol oxidase was determined by observing the conversion of the weakly fluorescent resazurin to the highly fluorescent resorufin and was normalized using crystal violet staining (indicating cell number). Mirroring the inhibitory activity observed in the cell-free model of CYP51 and the sterol profiles of treated cells, PC1244 strongly inhibited ergosterol production (IC_50_ = 0.0055 μg/ml; 0.0080 μM) and was 12-fold more potent than VRC (IC_50_ = 0.067 μg/ml; 0.19 μM) and 2.2-fold more potent than POS (IC_50_ = 0.012 μg/ml; 0.017 μM).

### *In vitro* determination of persistence of action.

**FIG 4:**
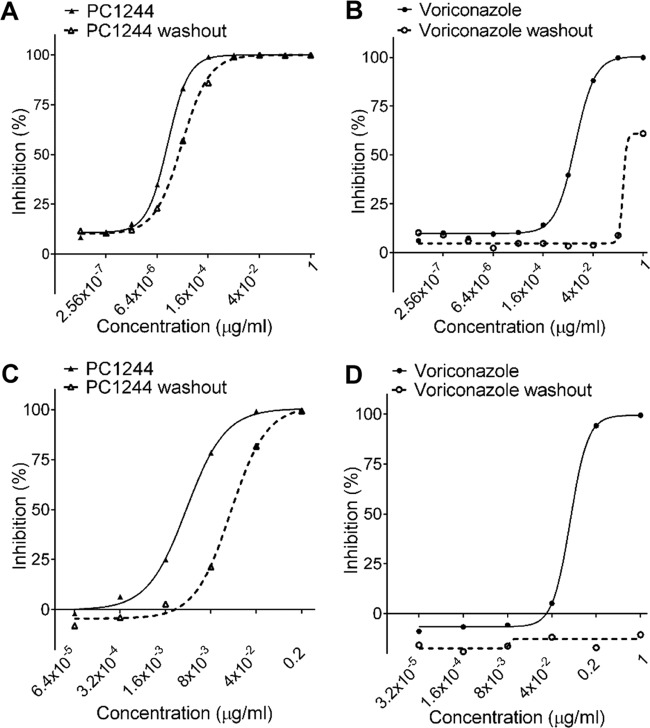
Duration of action of PC1244 against A. fumigatus. (A, B) Persistence of action of PC1244 (A) and voriconazole (B) on A. fumigatus hyphae. Values are means and SEMs from 3 independent experiments (each experiment was conducted in quadruplicate). (C, D) Persistence of action of PC1244 (C) and voriconazole (D) on human bronchial (BEAS2B) cells infected with A. fumigatus. Values are means and SEMs from three independent experiments (each experiment was conducted in triplicate).

The duration of action of test agents within the hyphae of A. fumigatus has been determined using a resazurin-based microtiter assay (28). A. fumigatus hyphae were exposed to the test agents for 16 h, the inhibition of fungal growth was measured, and the efficacy was compared with that obtained after contact with drug for only 20 min, followed by washout and incubation for the same period of time. As seen in Table 4, it was observed that PC1244 (IC_50_ = 0.00011 μg/ml) was 100- and 4.1-fold more potent than VRC and POS, respectively, at inhibiting hyphal A. fumigatus growth. In addition, the potency of VRC and POS diminished markedly, after short contact and washout, by factors of >93-fold and 4.90-fold, respectively. In contrast, washout produced only a 2.4-fold reduction in the activity of PC1244 compared with that with continuous contact in this experimental paradigm (Table 4; Fig. 4A and B).

**TABLE 4:** Potencies and persistence of action of PC1244, posaconazole, and voriconazole in A. fumigatus hyphae and in BEAS2B cells infected with A. fumigatus^*a*^

Test agent	Hyphae			BEAS2B cells		
	IC_50_ (μg/ml)		Fold change	IC_50_ (μg/ml)		Fold change
	No washout	Washout		No washout	Washout	
PC1244	0.00011^*b*^	0.00025	2.41	0.0034^*b*^	0.018	5.40
Voriconazole	0.011	>1	>93	0.054	>1	>18.6
Posaconazole	0.00045	0.0022	4.90	0.0031	0.046	14.7

a Data are from three independent experiments, and each assay was conducted in quadruplicate for the assay with hyphae and in triplicate for the assay with BEAS2B cells.

b *P* < 0.05 for PC1244 versus the results for voriconazole (Kruskal-Wallis one-way ANOVA with Dunn's test).

In a second system, the persistence of the action of the same three agents on A. fumigatus-infected bronchial epithelial cells was quantified using galactomannan (GM) production in the culture supernatant as an index of fungal growth. BEAS2B cells were infected with A. fumigatus, and the effects of a 24-h washout period (medium change) prior to infection were examined. A 1-h contact time with PC1244 followed by 24-h washout resulted in a 5.4-fold loss of potency against A. fumigatus compared with that achieved with the control, where there was no washout. POS showed a greater loss of its activity on washout (14.7-fold), and it was particularly notable that VRC was ineffective under the same test conditions (Table 4; Fig. 4C and D). The patterns of the effects mirror those seen in A. fumigatus hyphae (see above) and imply that only a short period of contact of bronchial epithelial cells with PC1244 would be required for the agent to exert a long duration of therapeutic action.

### *In vitro* fungicidal activity against A. fumigatus.

The minimum fungicidal concentration (MFC) for each compound was calculated 48 h after supernatants from the MIC assay were transferred to agar plates and was determined to be the lowest concentration of compound that yielded 3 colonies or less (CFU-MFC). PC1244 exhibited the greatest CFU-MFC of all compounds tested (2 μg/ml) and was 2- and 8-fold stronger than POS and VRC, respectively. The ratios of CFU-MFC versus MIC were 32, 32, and 9.6 for PC1244, POS, and VRC, respectively (Table 5).

**TABLE 5:** Mean fungicidal activity of PC1244, posaconazole, and voriconazole against A. fumigatus (NCPF2010)

Test agent	MIC or MFC (μg/ml) (MFC/MIC ratio)		
	MIC^*a*^	CFU-MFC^*b*^	XTT-MFC^*a*^
PC1244	0.063 ± 0	2 (32)	0.14 ± 0.0058 (2.2)
Voriconazole	1.67 ± 0.58	16 (9.6)	>32 (>19)
Posaconazole	0.125 ± 0	4 (32)	0.42 ± 0.020 (3.4)

a The values are the means from three biological replicates ± standard deviations of the means, and each assay was conducted in duplicate.

b The values are determined to be the lowest concentration to average less than 3 colonies per agar plate, across 3 independent experiments.

**FIG 5:**
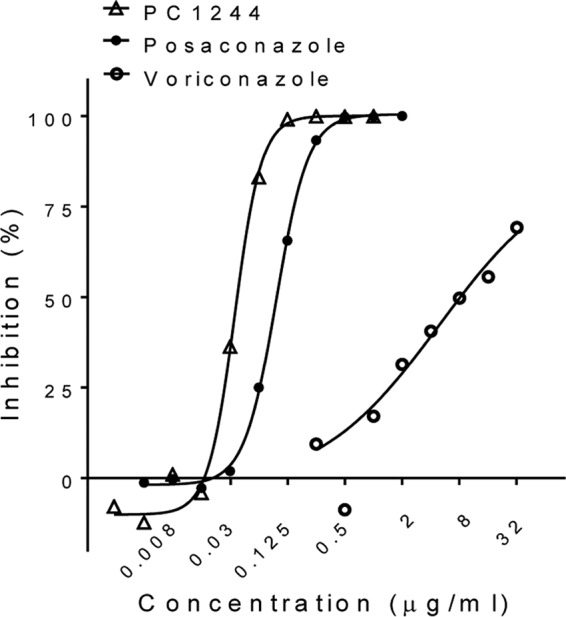
Colorimetric broth microdilution assessment of fungicidal activity of PC1244 against A. fumigatus (NCPF2010) *in vitro*. Values are means and SEMs from three independent experiments.

In addition, the fungicidal effect of each compound was determined using 2,3-bis-(2-methoxy-4-nitro-5-sulfophenyl)-2H-tetrazolium-5-carboxanilide salt (XTT)-based quantitative colorimetric analysis (XTT-MFC). The absorbance (OD) at 450 and 620 was measured 24 h after the supernatant including the compound was removed from the wells used for the broth microdilution MIC assay. Again, PC1244 exhibited the greatest level of inhibition of all compounds tested with an XTT-MFC of 0.14 μg/ml. In this system, PC1244 was 3-fold more potent than POS and >229-fold more potent than VRC. The maximum inhibition of fungicidal activity for PC1244, POS, and VRC was 99.9% at 1 μg/ml, 100% at 2 μg/ml, and 69.2% at 32 μg/ml, respectively. In addition, the ratios of the XTT-MFC to the MIC were 2.2, 3.4, and >19 for PC1244, POS, and VRC, respectively (Table 5; Fig. 5).

### *In vivo* antifungal activity on ITC-susceptible A. fumigatus infection.

**FIG 6:**
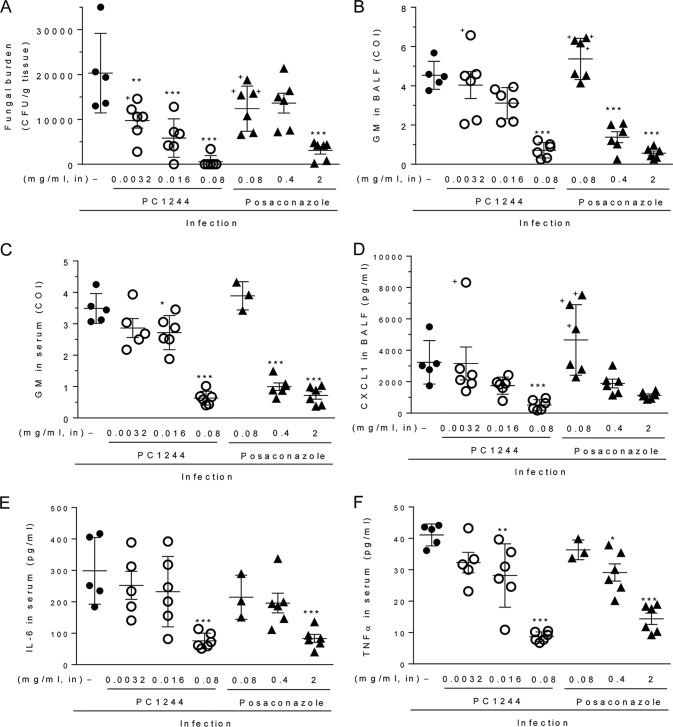
Antifungal activity of PC1244 against A. fumigatus *in vivo*. PC1244 (0.0032-, 0.0016-, and 0.08-mg/ml aqueous suspensions) and posaconazole (0.08-, 0.4-, and 2-mg/ml aqueous suspensions) were given intranasally (in) on days 1, 2, and 3 postinfection with A. fumigatus in temporarily neutropenic immunocompromised mice. The fungal load (number of CFU per gram of lung tissue) in lung (A), the galactomannan (GM) level in BALF (B), the GM level in serum (C), the CXCL1 concentration in BALF (D), the IL-6 concentration in serum (E), and tbe TNF-α concentration in serum (F) were evaluated on day 3 postinfection. Each horizontal bar presents the mean ± SD for 6 mice per group, except for nontreatment control (*n* = 5) and serum samples from posaconazole, 0.08 µg/ml (*n* = 3). *, *P* < 0.05 versus the infected control; **, *P* < 0.01 versus the infected control; ***, *P* < 0.001 versus the infected control. **+**, the mouse was dead before sample collection. Serum could not be collected from dead mice.

To assess the *in vivo* activity of PC1244, temporarily neutropenic mice infected with A. fumigatus (ATCC 13073 [strain NIH 5233]) were used. The MIC values of PC1244, POS, VRC, and ITC against this strain were 0.063, 0.125, 0.5, and 0.5 μg/ml, respectively. An aqueous suspension of PC1244 in isotonic saline (0.0032, 0.016, and 0.08 mg/ml, 35 μl; see Table 6 for the conversion to the number of milligrams per mouse or the approximate number of milligrams per kilogram of body weight) was dosed by intranasal injection once daily for 3 days postinfection with ITC-susceptible A. fumigatus. This late intervention regimen was found to strongly inhibit the fungal load (the number of CFU) in the lung, with the highest dose (0.08 mg/ml) administered exhibiting 97% inhibition compared to that achieved with the vehicle (Fig. 6A). In comparison, POS, given at the same level of 0.08 mg/ml, achieved only 39% inhibition of the lung fungal load, and the dose required to reduce the load by 1 log_10_ CFU/g (the log_10_ inhibitory dose [ID] value) was 2.0 mg/ml (70 μg/mouse), which was 143-fold higher than that of PC1244 (log_10_ ID, 0.014 mg/ml [0.49 μg/mouse]). PC1244 also decreased the GM concentrations in bronchoalveolar lavage fluid (BALF) in a dose-dependent manner, showing a 50% inhibitory dose (ID_50_) value of 0.032 mg/ml (1.1 μg/mouse), which was 6.7-fold lower than that of POS (ID_50_, 0.21 mg/ml [7.4 μg/mouse]) (Fig. 6B). PC1244 decreased the GM concentrations in serum in a dose-dependent manner, too (Fig. 4C). Notably, 0.08 mg/ml (2.8 μg/mouse) of PC1244 produced marked inhibition (82% inhibition) of GM in serum, whereas POS, at the same dose, did not show any effect (−11% inhibition). In a pilot study, we also measured A. fumigatus PCR products in lung tissue, and consistent with the data presented above, PC1244 inhibited the accumulation of PCR products (see Fig. S1B in the supplemental material). In addition, PC1244 also reduced the A. fumigatus infection-dependent increase in the CXCL1 level in BALF (Fig. 6D) and the interleukin-6 (IL-6) (Fig. 6E) and tumor necrosis factor alpha (TNF-α) (Fig. 6F) levels in serum.

**TABLE 6:** Conversion of units of dose given to mice in *in vivo* study

Concn (mg/ml) in aqueous suspension	Concn as mg/mouse	Approximate concn as mg/kg^*a*^
0.0032	0.00011	0.0056
0.016	0.00056	0.028
0.08	0.0028	0.14
0.4	0.014	0.70
0.8	0.028	1.4
2	0.07	3.5

a For calculation, 20 g was used as the average body weight of the mice.

**FIG 7:**
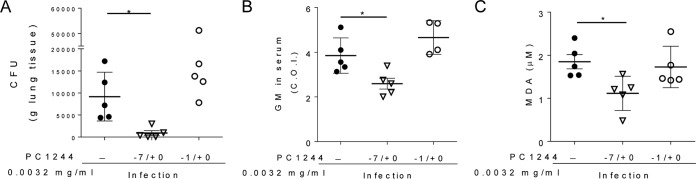
Antifungal activity of PC1244 against A. fumigatus with extended prophylactic treatment *in vivo*. The effect of 7 days of extended prophylaxis with intranasal PC1244 on the lung fungal load (number of CFU per gram of tissue) (A), the GM level (COI) in serum (B), and the malondialdehyde (MDA) concentration in BALF (C) of A. fumigatus-infected immunocompromised mice (*n* = 4 or 5) was compared with that of only 1 day of prophylactic treatment. Each horizontal bar presents the mean ± SD for 4 to 6 mice per group. *, *P* < 0.05.

Extended prophylaxis with PC1244 (0.0032 mg/ml [0.11 μg/mouse]; 7 days of extended prophylaxis and Aspergillus inoculation on day 0 [−7/0]) achieved high levels of inhibitory effects on the fungal load and biomarkers compared to those observed in the late intervention study (treatment on days 1 to 3 postinfection) at the same dose (Fig. 6A versus 7A and 6C versus 7B). Furthermore, a marked difference between extended prophylaxis (−7/0) and a shorter period of prophylactic treatment (1 day of prophylaxis and Aspergillus inoculation on day 0 [−1/0]) was also observed for the number of CFU in lung, the level of galactomannan in serum, and the level of malondialdehyde (MDA; an oxidative stress marker) in BALF (Fig. 7A to C).

**FIG 8:**
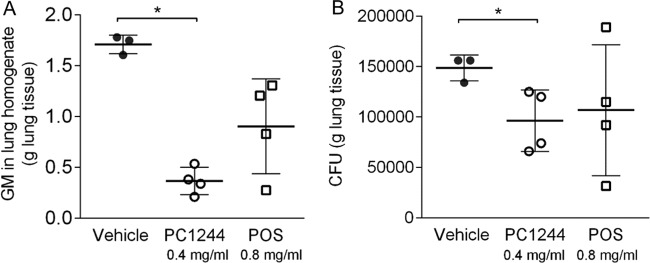
Effect of single prophylactic treatment with PC1244 and posaconazole against A. fumigatus *in vivo*. PC1244 at 0.4 mg/ml (14 μg/mouse) and posaconazole at 0.8 mg/ml (28 μg/mouse) were intranasally administered 16 h before A. fumigatus inoculation, and the lungs were collected for galactomannan (GM) level (COI) (A) and fungal load (number of CFU per gram of lung tissue) (B) assessment at 8 h after A. fumigatus inoculation. Each bar presents the mean ± SD for 3 to 4 mice per group. *, *P* < 0.05.

As we indicated earlier that the action of PC1244 in the *in vitro* system was persistent, the persistence of action was also evaluated *in vivo*. As the MIC value for the A. fumigatus strain (ATCC 13073) used in this study was 2-fold lower for PC1244 than for POS, PC1244 at 0.4 mg/ml (14 μg/mouse) and POS at 0.8 mg/ml (28 μg/mouse) were intranasally administered 16 h before A. fumigatus inoculation, and the lungs were collected 8 h after A. fumigatus inoculation for assessment of GM levels and the number of CFU. As observed in Fig. 8A and B, PC1244 showed significant inhibition of both GM levels and the number of CFU in the lung, but POS did not, despite its administration at a 2-fold higher dose. Thus, in the *in vivo* system, the persistent action of PC1244 was confirmed.

### Antifungal activity against non-A. fumigatus species.

The *in vitro* activity of PC1244 was compared with that of VRC and POS against 23 pathogenic fungi (1 to 2 isolates each), and the results are displayed in Table 7. In all non-Aspergillus and non-Candida species tested, PC1244 was more potent than POS and VRC or had potency comparable to that of POS and VRC. Of particular note, PC1244 was effective (MIC, 0.25 to 2 μg/ml) against species against which VRC and POS had no effect within the concentration range tested (MIC, >8 μg/ml), including Gibberella zeae (Fusarium graminearum), Lichtheimia corymbifera, Mucor circinelloides, Rhizomucor pusillus, and Rhizopus oryzae. PC1244 was found to have antifungal activity against Aspergillus flavus, Aspergillus niger, and Aspergillus terreus, albeit with less potency than POS. Against Aspergillus carbonarius, PC1244 was as potent as POS and more potent than VRC. Against all Candida species tested (Candida albicans, Candida glabrata, Candida krusei, and Candida parapsilosis), PC1244 was more potent than VRC and was stronger than POS or had inhibitory activity comparable to that of POS.

**TABLE 7:** Antifungal effects of PC1244, voriconazole, and posaconazole on other fungal species

Species [strain(s)]	No. of strains tested	Culture method	MIC (μg/ml)^*a*^		
			PC1244	Voriconazole	Posaconazole
Aspergillus carbonarius (ATCC 8740)^*d*^	1	CLSI	0.063	0.5	0.063
Aspergillus flavus (ATCC 204304)^*d*^	1	CLSI	0.25	2	0.13
Aspergillus flavus (AFL8, NRRC3357)	2	EUCAST	0.38	0.63	0.16
Aspergillus niger (ATCC 1015)	1	EUCAST	0.5	1	0.20
Aspergillus terreus (AT49, AT7130)	2	EUCAST	0.38	1	0.093
Penicillium chrysogenum (ATCC 9480)^*d*^	1	CLSI	0.13	2	0.13
Penicillium citrinum (ATCC 9849)^*d*^	1	CLSI	0.5	>8	0.5
Trichophyton rubrum (ATCC 10218)^*d*^	1	CLSI	0.031	0.063	0.031
Aureobasidium pullulans (ATCC 348)^*d*^	1	CLSI	1	>8	1
Cladosporium argillaceum (ATCC 38013)^*d*^	1	CLSI	0.25	0.5	0.25
Candida albicans (20240.047, ATCC 10231)^*d*^	2	CLSI	<0.0078^*b*^	0.14^*b*^	0.081^*b*^
Candida albicans AR^*c*^ (20183.073, 20186.025)^*d*^	2	CLSI	(0.25, <0.0078)^*b*^^,^^*f*^	10^*b*^	8.13^*b*^
Candida glabrata (ATCC 36583, R363)^*d*^	2	CLSI	(<0.0078, 0.13)^*b*^^,^^*f*^	8.13^*b*^	0.5^*b*^
Candida krusei (ATCC 6258)^*d*^	1	CLSI	0.13	0.25	0.125
Candida parapsilosis (ATCC 22019)^*d*^	1	CLSI	0.25^*b*^	NT^*e*^	0.25^*b*^
Chaetomium globosum (ATCC 44699)^*d*^	1	CLSI	0.063	1	0.25
Gibberella zeae(Fusarium graminearum) (ATCC 16106)^*d*^	1	CLSI	1	>8	>8
Cryptococcus gattii (clinical isolate)	1	EUCAST	0.5	0.125	0.5
Cryptococcus neoformans (ATCC 24067)^*d*^	1	CLSI	0.016	0.016	0.016
Lichtheimia corymbifera (ATCC 7909)^*d*^	1	CLSI	1	>8	>8
Mucor circinelloides (ATCC 8542)^*d*^	1	CLSI	2	>8	>8
Rhizomucor pusillus (ATCC 16458)^*d*^	1	CLSI	2	>8	>8
Rhizopus oryzae(ATCC 11145)^*d*^	1	CLSI	0.25	>8	>8

a Due to the limited number of strains tested, the mean MICs for the isolates are presented.

b The MIC indicates 50% inhibition of fungal growth as the readout for the azoles.

c AR, azole (fluconazole and voriconazole) resistant.

d All details for the isolates and the assay protocol are described by Eurofins Panlabs, Inc., (anti-infective assay/fungi).

e NT, not tested.

f Two individual MIC values are shown as it shows different susceptibilities.

## DISCUSSION

In this report, we present data demonstrating that (i) the novel triazole PC1244 possesses both potent and persistent antifungal activity and significant fungicidal activity against ITC-susceptible and/or ITC-resistant A. fumigatus *in vitro*, (ii) the antifungal activity was confirmed in clinical isolates from two geographical areas, (iii) intranasal once-daily PC1244 treatment exhibits potent antifungal effects against A. fumigatus *in vivo* in temporarily neutropenic mice, and (iv) PC1244 shows a broad range of antifungal activity when screened against a panel of pathogenic fungal organisms.

The proposed mechanism of action of PC1244 is inhibition of sterol 14α-demethylase (CYP51A1), the enzyme required to convert eburicol to 14-demethylated eburicol, an essential step in the ergosterol biosynthesis pathway in fungi. Type II binding spectra, which display a maximum absorbance at 423 to 430 nm and a broad trough absorbance at 386 to 412 nm, arise through a specific interaction in which the triazole (posaconazole) N-4 nitrogen or the imidazole ring N-3 nitrogen coordinates as the sixth axial ligand with the heme iron to form a low-spin CYP51-azole complex (29, 30). PC1244 produced type II difference spectra when titrated against purified recombinant ITC-susceptible A. fumigatus (AF293) CYP51A and CYP51B and bound to both enzymes with an affinity similar to that of posaconazole. Furthermore, the strong inhibition of CYP51A activity observed with both PC1244 and posaconazole, characteristic of tight-binding inhibitors (IC_50_, approximately half that of the enzyme concentration present), exceeded that predicted by the *K_d_* values calculated from ligand binding studies using recombinant CYP51A, suggesting that the conformation of purified CYP51A in solution differs from that in cell membranes.

In sterol composition determinations, treatment with increasing concentrations of either PC1244 or posaconazole ranging from 0 to 1 μg/ml resulted in an accumulation of the 14-methylated sterols lanosterol and eburicol and depletion of the final sterol product, ergosterol; this pattern of effect is consistent with CYP51 inhibition being the key pharmacological activity of both agents. In addition, a cell-based assay of ergosterol biosynthesis in A. fumigatus demonstrated that PC1244 was 12- and 2.2-fold more potent at inhibiting ergosterol production than voriconazole and posaconazole, respectively. Thus, the mechanism of action of PC1244, as for other triazole antifungals, is the inhibition of fungal sterol 14α-demethylase, resulting in the depletion of ergosterol in the fungal membrane, so disrupting the membrane structure and function and inhibiting the growth of the pathogenic organism (31).

A highly desirable feature of topical medicines is a long duration of action, ensuring that the desired therapeutic activity is maintained throughout the interdose period. This is particularly relevant to the treatment of pulmonary infection with A. fumigatus, which germinates in both extracellular environments and intracellular compartments. The duration of action of PC1244 was therefore considered an important property and was evaluated in a variety of *in vitro* systems. In A. fumigatus hyphae, the IC_50_ measured for PC1244 following a 20-min contact period and washout for 16 h was reduced only 2.4-fold relative to that obtained following continuous contact with the drug for the same period without washout. Furthermore, in the BEAS2B cell line, washout for 24 h, after a 1-h contact period, resulted in only an approximately 5-fold loss of potency against A. fumigatus compared with that of the control. These observed properties of rapid cellular penetration and persistence of action in the absence of the pathogen may be particularly valuable characteristics which enhance the potential use of PC1244 in prophylaxis. The persistent action of PC1244 was also confirmed in the *in vivo* system when administered 16 h before A. fumigatus inoculation (Fig. 5).

In the *in vivo* system, intranasal treatment with PC1244 showed that it had better effects than posaconazole, despite comparable MIC values in *in vitro* testing. We speculate, first, that the persistence of the drug substance on bronchial cells, as shown in Fig. 4, is likely a factor contributing to the amplification of the antifungal effects of PC1244 *in vivo* seen in the current once-daily treatment regimen. Second, we have demonstrated here that a 7-day extended prophylactic treatment (using very low doses) produced much greater anti-Aspergillus activity than prophylactic treatment for 1 day and also that the effects of a 7-day prophylactic treatment were maintained if treatment ceased when Aspergillus was inoculated on day 0 (Fig. 7). This is powerful pharmacodynamic evidence that the effects of PC1244 accumulate on daily dosing in mice and are maintained when dosing is terminated. Third, Baistrocchi and colleagues published evidence of the accumulation of posaconazole in granulocyte-type cells and demonstrated enhanced synergic antifungal effects (by exposure of Aspergillus to cellular posaconazole during phagocytosis) (32). Considering the persistent action of PC1244, it is likely that granulocytes/macrophages containing PC1244 contributed to further enhancement of the antifungal effect.

Determining whether an antifungal compound is fungistatic or fungicidal is complex, and the clinical utility of such a characterization is the subject of much debate. The treatment of fungal infections of body compartments that are not easily accessed by host defenses requires agents that are fungicidal in nature, and this is especially true in immunocompromised patients (33). For an antibiotic to qualify as bactericidal, its minimum bactericidal concentration (MBC) must be no more than 2× to 4× the MIC, but the definition of “fungicidal” is yet to be standardized (33). Determination of MFCs for filamentous fungi is not standardized either, but studies have shown that reproducible MFCs can be obtained by following standardized broth microdilution methods for MIC determination, followed by subculture onto agar (33, 34). Defining an MFC to be the lowest drug dilution to yield less than 3 colonies to obtain 99% to 99.5% killing activity, Espinel-Ingroff et al. determined the range of MFCs for inhibition of 90% of isolates tested (MFC_90_) for itraconazole (0.2 to 4 μg/ml), voriconazole (0.5 to 4 μg/ml), and posaconazole (0.06 to 2 μg/ml) for a number of A. fumigatus isolates (34). However, it is worth noting that the agar subculture methodology tests for fungicidal activity on planktonic A. fumigatus growth only. Here we used a combination of different methodologies to attempt to determine the fungicidal activity of PC1244 and clinically used triazoles accurately. The use of subculture on agar (CFU-MFC) gave an MFC of 2 μg/ml for PC1244 with an MFC/MIC ratio of 32, and similar results were seen with posaconazole (MFC = 4 μg/ml, MFC/MIC = 32), while voriconazole exhibited a higher MFC (16 μg/ml) but a lower MFC/MIC ratio (9.6). As discussed above, these data would suggest that voriconazole is a more fungicidal compound, as it exhibits a lower MFC/MIC ratio. However, recent studies have shown that this technique likely overestimates the fungicidal activity of a compound, as it does not factor in viable conidia attached to the base of test wells (35). To account for this phenomenon, a colorimetric method for assessing the fungicidal activity of a compound against sessile A. fumigatus was used (35). MFC determination by this microbroth colorimetric method (XTT-MFC) gave an MFC of 0.14 μg/ml for PC1244 with an MFC/MIC ratio of 2.2, which were superior to the values for both posaconazole (MFC = 0.42 μg/ml, MFC/MIC = 3.4) and voriconazole (MFC = >32 μg/ml, MFC/MIC = >19). Therefore, with these data we provide evidence that PC1244 is a fungicidal compound with a degree of potency similar to that of posaconazole or improved over that of posaconazole.

As with any study there are limitations, and this was especially the case for the *in vivo* study. First, the delivery system does not mimic clinical use. The advantage of intranasal instillation is being able to confirm that all the solution is delivered into the body, but we did not control the level of lung exposure or the exposure site (the same as aerosolization). However, we carefully optimized the intranasal dosing volume, as it has been shown that approximately 60% of the administered dose is deposited in the lung after intranasal treatment (36), and we also confirmed trachea/lung deposition after intranasal administration of 35 μl of methylene blue solution to A/J mice (data not shown). Aerosol delivery with close drug monitoring at the exposure site (rather than systemic delivery) is ideal, but this is not easily achieved, as special imaging equipment is required, as Miller et al. elegantly demonstrated using whole-animal luminescent imaging (37). Second, the numbers of CFU were determined only in the whole left lobe. This causes location bias of the fungal load, and ideally, we should test the numbers of CFU in a homogenate from the whole lung. However, when we determined the numbers of CFU and the GM levels in the right lobe and the left lobe, we did not find any significant difference of these biomarkers between the right lobe and the left lobe (data not shown). In addition, to avoid this bias, we determined GM levels in BALF and serum. Third, there was a lack of pharmacokinetic measurement of PC1244 in the mice used for the *in vivo* study. PC1244 has been optimized for topical treatment of the lung to maximize local exposure and minimize systemic exposure. Systemic concentrations of drug are therefore not a useful surrogate marker to help explain the different antifungal efficacies of compounds. However, we have some data demonstrating that measurable levels of systemic exposure do occur. In preliminary studies using noninfected mice dosed intratracheally with 40 μl of a 2-mg/ml aqueous suspension of PC1244, it was shown that the plasma concentrations of PC1244 ranged from 111 to 303 ng/ml at 2 h postdose and 249 to 339 ng/ml at 8 h postdose, but this was reduced to 41.5 to 50.7 ng/ml at 24 h postdose, despite decent *in vivo* effects after once-daily treatment. Under the same conditions, the plasma concentrations achieved with posaconazole were 15.6 to 125 ng/ml at 24 h postdose. These concentrations were more variable but not too different from those of PC1244, although the *in vivo* activity of PC1244 was superior to that of posaconazole. As PC1244 has much less oral availability than posaconazole (unpublished data), the exposure results from absorption through the respiratory tract (not by accidental ingestion of compound during dosing). Furthermore, all compounds are water insoluble and administered topically (where the respiratory tract is exposed directly). We do not, therefore, believe that water solubility is a key factor explaining the *in vivo* efficacies or topical exposure levels. Finally, we tested only limited isolates of azole-resistant strains. Further study with a wide range of clinical azole-resistant isolates with different genotypes will be required to determine the potency of PC1244 against recent A. fumigatus strains with TR34/L98H and TR46/Y121F/T289A mutations or some other genetic cause(s) underlying resistance.

Thus, due to its activity that is superior to that of voriconazole or comparable to that of posaconazole against both azole-susceptible and azole-resistant A. fumigatus strains, persistent action, extended retention within the lung after topical treatment, and broad repertoire of fungal targets, PC1244 has the potential to become a valuable new therapeutic agent for the treatment of A. fumigatus and other difficult-to-treat fungal infections in humans.

## MATERIALS AND METHODS

### Antifungal agents.

PC1244 was synthesized by Sygnature Discovery Ltd. (Nottingham, UK), and voriconazole (Tokyo Chemical Industry UK Ltd., Oxford, UK), posaconazole (Apichem Chemical Technology Co., Ltd., Zhejiang, China), itraconazole (Arkopharma, Carros, France), amphotericin B (Selleckchem, Munich, Germany), and caspofungin (Selleckchem, Munich, Germany) were procured from commercial sources. For *in vitro* antifungal assays, stock solutions of the test agents were prepared in dimethyl sulfoxide (DMSO; 2,000 μg/ml). For *in vivo* studies, solid materials of the test agents were directly suspended in physiological saline at 10 mg/ml and diluted with physiological saline after sonication.

### A. fumigatus CYP51 binding assay and enzyme-inhibitory activity.

A. fumigatus CYP51 binding properties were determined as previously reported (28, 38). Test agents were titrated against 4 μM recombinant A. fumigatus (AF293 strain) CYP51A or CYP51B proteins, and binding saturation curves were constructed from the change in the absorbance between the spectral peak and the trough. A rearrangement of the Morrison equation was used to determine the dissociation constant (*K_d_*) values when ligand binding was tight (39).

A CYP51 reconstitution assay system was used to determine 50% inhibitory concentrations (IC_50_) (40). The test agent was added to a mixture of 0.5 μM CYP51, 1 μM A. fumigatus cytochrome P450 reductase isoenzyme 1 (AfCPR1), 50 μM eburicol, 4% (wt/vol) 2-hydroxypropyl-β-cyclodextrin, 0.4 mg/ml isocitrate dehydrogenase, 25 mM trisodium isocitrate, 50 mM NaCl, 5 mM MgCl_2_, and 40 mM 3-(*N*-morpholino) propanesulfonic acid (MOPS; pH of ∼7.2). The mixtures were then incubated at 37°C for 10 min prior to initiation with 4 mM β-NADPHNa_4_, followed by shaking for 20 min at 37°C. Sterol metabolites were recovered by extraction with ethyl acetate followed by derivatization with 0.1 ml *N*,*O*-bis(trimethylsilyl)trifluoroacetamide: trimethylchlorosilane (99:1) and 0.3 ml anhydrous pyridine prior to analysis by gas chromatography-mass spectrometry.

### A. fumigatus sterol analysis.

A working suspension of A. fumigatus spores (NCPF2010) was prepared in filter-sterilized MOPS-RPMI 1640 (RPMI 1640 containing 2 mM l-glutamine, 2% glucose, and 0.165 M MOPS buffered to pH 7 with NaOH) at a final concentration of 8 × 10^6^ spores ml^−1^. To each 100-mm petri dish, 10 ml of the working suspension was added, and the dishes were incubated for 4 h at 35°C in 5% CO_2_. Samples for baseline determinations were collected by scraping, pelleted by centrifugation at 2,000 rpm for 5 min, and stored at −80°C. Test compounds or DMSO (50 μl) was added to the remaining dishes, which were subsequently gently rocked by hand to disperse the compounds. The dishes were incubated for 2 h at 35°C in 5% CO_2_. Samples were collected and processed as described above. Posaconazole and PC1244 concentrations of 0.0001, 0.001, 0.01, 0.1, and 1 μg ml^−1^ were tested. These samples were prepared in the laboratory at Pulmocide Ltd. and sent to the laboratory in the Centre for Cytochrome P450 Biodiversity, Institute of Life Science, Swansea University Medical School, for analysis.

Nonsaponifiable lipids were extracted as previously reported (31) and were derivatized with 0.1 ml *N*,*O*-bis(trimethylsilyl)trifluoroacetamide–trimethylchlorosilane (99:1) and 0.3 ml anhydrous pyridine (2 h at 80°C), prior to analysis by gas chromatography-mass spectrometry (41). The sterol composition was calculated using peak areas from the gas chromatograms, and mass fragmentation patterns compared to those of known standards were used to confirm sterol identity. The sterol contents of A. fumigatus (basal) and treated A. fumigatus (treated with either DMSO, posaconazole, or PC1244) were determined in three biological replicates.

### A. fumigatus cell-based ergosterol assay.

Growth medium (RPMI 1640, 2 mM l-glutamine, 2% glucose, 0.165 M MOPS, 0.5% bovine serum albumin [BSA], pH 7.0) was added across a 96-well plate and the test agents were added in duplicate. A. fumigatus (NCPF2010) conidia were added across the plate at a final concentration of 1 × 10^4^ ml^−1^. After incubation for 24 h at 35°C, the medium was removed from all wells and replaced with reaction buffer (catalog number A12216; Amplex red cholesterol assay kit; Thermo Fisher) and Amplex red solution. The plates were incubated for 30 min at 37°C with protection from light, after which the fluorescence was quantified using a spectrophotometer. The medium was removed from all wells and replaced with crystal violet solution (1%, vol/vol), and the plates were incubated at room temperature on a shaker for 30 min. The plates were washed three times with phosphate-buffered saline (PBS), and sodium dodecyl sulfate solution (0.1%, vol/vol) was added across the plate to lyse the cells. After incubation at room temperature for 1 h, the absorbance (OD) at 590 nm was measured using a spectrophotometer.

### *In vitro* antifungal activity against A. fumigatus.

Assessment of antifungal activity against a selection of A. fumigatus laboratory/clinical strains (NCPF2010, AF72, AF91, AF293, and AF294, all from the National Collection of Pathogenic Fungi [NCPF], Bristol, UK) was performed using the EUCAST methodology, as previously reported (28), in a 384-well plate format as quadruplicates in three independent experiments. Growth medium (RPMI 1640, 2 mM l-glutamine, 2% glucose, 0.165 M MOPS, 0.5% BSA, pH 7.0) was added across the plate, test agents were added in quadruplicate, and the DMSO concentration was identical across the plates. Conidia were added across the plate at a final concentration of 1 × 10^5^ ml^−1^. The plates were incubated for 48 h at 35°C, after which the turbidity was assessed by measuring the optical density (OD) at 530 nm using a spectrophotometer, and the IC_50_ and IC_90_ values were calculated from the concentration-response curve generated for each test compound using a four-parameter logistic equation (Dotmatics, Bishops Stortford, UK). A. fumigatus ATCC 204305 was used as the assay control. Determination of antifungal activity against 50 A. fumigatus clinical isolates from Saint Louis Hospital (Paris, France) was performed with 96-well plates using the EUCAST method described above (28) in duplicate. Antifungal susceptibility testing for 46 A. fumigatus isolates (obtained from the North West England Mycology Reference Centre) was performed for each isolate by Evotec (UK) Ltd. (Manchester, UK) according to EUCAST guidelines. Assessment of the antifungal activity against each of four A. fumigatus strains (ATCC 1028, ATCC 10894, ATCC 13073, and ATCC 16424) was performed according to the M38-A methodology described by the Clinical and Laboratory Standards Institute (CLSI) (26) by Eurofins Panlabs, Inc. (Taipei, Taiwan).

### *In vitro* antifungal activity against other fungal species.

For the measurement of activity against C. gattii, the method described in EUCAST definitive document EDef 7.2 was used, and the assay plates were incubated statically at 37°C in ambient air for 24 ± 2 h, unless poor growth necessitated further incubation to 36 or 48 h (42). Antifungal potency against Aspergillus flavus, Aspergillus niger, and Aspergillus terreus was determined as set out in EUCAST definitive document EDef 9.2, and the assay plates were incubated at 37°C for 48 h (43). These tests were conducted at Evotec (UK) Ltd. (Manchester, UK). Measurement of the activity against other fungi was performed by Eurofins Panlabs, Inc., according to the methodology described by the Clinical and Laboratory Standards Institute (CLSI; CLSI M38-A [26] or M27-A2 [44]). The source or strain name of each fungus species is indicated in Table 7. The MICs against C. albicans, C. parapsilosis, and C. glabrata were determined using an azole endpoint which indicates 50% inhibition of fungus growth.

### *In vitro* fungicidal activity of PC1244 against A. fumigatus.

The antifungal activity of PC1244 against A. fumigatus (NCPF2010) was determined in 96-well plates, using the methodology described above, as duplicates in three independent experiments. After the MIC for each compound was recorded, fungicidal activity was determined as previously described (35). Briefly, medium from each well (100 μl/well) was removed after pipetting up and down five times and subcultured onto 4% Sabouraud dextrose agar plates. The plates were incubated (35°C with ambient air) for 48 h, and the numbers of CFU were counted for each compound concentration. The minimum fungicidal concentration (MFC) was determined as the lowest compound concentration yielding 3 colonies or less.

After the removal of medium for culture-based testing of the number of CFU, the contents of all wells were carefully aspirated and warm PBS (200 μl/well) was added. After gentle agitation, the contents of all wells were aspirated and fresh medium was added (200 μl/well). The plates were incubated (35°C with ambient air) for 24 h. A working solution of 0.5 mg/ml XTT and 125 μM menadione was prepared in PBS and added across the plate (50 μl/well). The plates were incubated (35°C with ambient air) for 2 h, after which the plates were agitated gently for 2 min. The optical density (OD) of each well at 450 nm and 620 nm was measured using a multiscanner (Clariostar; BMG, Buckinghamshire, UK). The MFC (XTT-MFC) was calculated from the concentration-response curve generated using a cutoff of 99% inhibition.

### *In vitro* determination of persistence of action on A. fumigatus hyphae.

The persistence of action of the test agents against A. fumigatus hyphae (NCPF2010) was determined as previously reported (28). Briefly, conidia diluted in growth medium (RPMI 1640, 2 mM l-glutamine, 2% glucose, 0.165 M MOPS, pH 7.0) were added across a 384-well plate at a final concentration of 1 × 10^3^/well. After incubation at 35°C for exactly 6 h, test and reference articles or neat DMSO (as the vehicle) (0.5 μl/well) was added to the appropriate wells to give a final concentration of 0.5% DMSO. The plates were incubated for exactly 20 min at 35°C in 5% CO_2_. After the incubation time had elapsed, all wells on the designated washout plate were aspirated and growth medium (100 μl/well) was added across the plate. For the nonwashout plate, after the compounds were added to the hyphae, no medium change was applied. Resazurin (0.04% diluted in growth medium) was added to all wells of both the nonwashout and washout plates (5 μl/well) to give a final concentration of 0.002% resazurin. The plates were incubated at 35°C in 5% CO_2_ for 16 h. Subsequently, the fluorescence in each well was measured at an excitation λ of 545 nm and an emission λ of 600 nm using a multiscanner (Clariostar; BMG, Buckinghamshire, UK). The percent inhibition for each well was calculated, and the IC_50_ was calculated from the concentration-response curve generated for each test compound using a four-parameter logistic equation (Dotmatics, Bishops Stortford, UK). This study was conducted in quadruplicate in three independent experiments.

### *In vitro* determination of persistence of action in bronchial epithelial cells.

The persistence of action of the test agents was evaluated in immortalized bronchial epithelial cells (BEAS2B cells) as previously reported (28). Each experiment consisted of one nonwashout plate (96-well) and a parallel washout plate into which BEAS2B cells were seeded at a concentration of 3 × 10^4^ cells/well in growth medium (RPMI 1640, 2 mM l-glutamine, 10% fetal calf serum), and the plates were incubated for 24 h at 37°C in 5% CO_2_. Test and reference articles or neat DMSO (as the vehicle) (0.5 μl/well) was added to the appropriate wells of the washout plate to give a final concentration of 0.5% DMSO. The plate was incubated for exactly 1 h at 37°C in 5% CO_2_. After the incubation time had elapsed, all wells on the washout plate were aspirated and growth medium (100 μl/well) was added across the plate. After 24 h of incubation at 37°C, test and reference articles or neat DMSO (as the vehicle) (0.5 μl/well) was added to the appropriate wells of the nonwashout plate to give a final concentration of 0.5% DMSO. The plate was incubated for exactly 1 h at 37°C in 5% CO_2_, after which A. fumigatus conidia were added across both plates at a final concentration of 1 × 10^3^/well. Fungal growth was determined after a further 24 h of incubation at 35°C in 5% CO_2_ by measuring the galactomannan (GM) concentrations using Platelia GM enzyme immunoassay (EIA) kits (catalog number 62794; Bio-Rad Laboratories). The percent inhibition for each well was calculated, and the IC_50_ was calculated from the concentration-response curve generated for each test compound using a four-parameter logistic equation (Dotmatics, Bishops Stortford, UK). This study was conducted in triplicate in three independent experiments.

### *In vivo* antifungal activity against A. fumigatus infection.

As previously reported (28), we tested the antifungal effects of the test articles on A. fumigatus-infected, temporarily neutropenic mice. Specific-pathogen-free A/J mice (male, 5 weeks old) were used for A. fumigatus infection, as they have previously been described to be more susceptible to A. fumigatus infection (45). Animals (*n* = 6 per group) were then dosed with hydrocortisone (125 mg/kg of body weight subcutaneously; catalog number H4881; Sigma) on days 3, 2, and 1 before infection and with cyclophosphamide (250 mg/kg intraperitoneally; catalog number C0768; Sigma) 2 days before infection to induce temporary neutropenia. Both hydrocortisone and cyclophosphamide were diluted with physiological saline. To avoid bacterial infection during immunosuppression, the drinking water was supplemented with tetracycline hydrochloride (1 μg/ml; catalog number T7660; Sigma) and ciprofloxacin (64 μg/ml; catalog number 17850; Fluka). Conidia of A. fumigatus (ATCC 13073 [strain NIH 5233]) were aseptically dislodged from the malt agar plates and suspended in sterile distilled water with 0.05% Tween 80 and 0.1% agar. On the day of infection, 30 μl (15 μl in each nostril) of the conidial suspension (1.67 × 10^8^/ml in physiological saline) was administered intranasally while the mice were under 3% isoflurane anesthesia.

The test agents, suspended in physiological saline, were administered intranasally (35 μl, approximately 17.5 μl each in each nostril) once daily on days 1, 2, and 3 postinfection. To investigate extended prophylaxis, PC1244 was administered intranasally once daily on days −7 to 0, and the effects were compared with those of treatment on days −1 to 0. As the injection volume was fixed and the body weight changed every day, especially after infection, the accurate dose unit was the number of micrograms per mouse. However, as the average body weight after immunosuppression and just before infection was 20 g, we also calculated the estimated dose in milligrams per kilogram. Therefore, 35-μl injections of 0.0032, 0.016, 0.08, 0.4, and 2 mg/ml were equivalent to 0.11, 0.56, 2.8, 14, and 70 μg/mouse, respectively, which were equivalent to approximately 0.0056, 0.028, 0.14, 0.7, and 3.5 mg/kg, respectively (Table 6).

Both a body weight loss of >20% compared with the animal's weight on day 1 and mouse death were defined as dropout events. Animals that lost >20% of their initial body weight were sacrificed. Animals were terminally anesthetized 6 h after the last dose of drug was administered on day 3. The volume inserted intranasally is reported to achieve almost 60% deposition into the lung (36).

BALF was collected through cannulated tracheas using physiological saline (46). Blood was then collected via cardiac puncture, and lung tissue was removed for homogenate preparation. The Aspergillus GM concentration in serum was determined with Platelia GM EIA kits (catalog number 62794; Bio-Rad Laboratories). The value was provided as a cutoff index (COI), which was calculated by the formula COI = OD in sample/OD in cutoff control provided by the kit. For tissue fungal load, 100 mg of the whole left lobe of lung tissue was removed aseptically and homogenized in 0.2 ml of 0.1% agar in sterile distilled water as previously reported (28). We confirmed that the number of CFU was not significantly different between the right lung and left lung. Serially diluted lung homogenates were plated on malt agar plates (50 μl/plate), and the plates were incubated at 24 ± 1°C for 72 to 96 h. The colonies of A. fumigatus on each plate were counted, and the fungal titer is presented here as the number of CFU (×10^3^) per gram of lung tissue.

Measurement of TNF-α and IL-6 levels in serum and the CXCL1 level in BALF was performed using a Quantikine mouse enzyme-linked immunosorbent assay kit (R&D Systems, Inc., Minneapolis, MN, USA). Malondialdehyde (MDA) analysis was also performed using an OxiSelect thiobarbituric acid-reactive substances assay kit (MDA Quantitation; Cell Biolabs Inc., San Diego, CA, USA). For quantitative PCR, DNA amplification was performed with Premix *Ex Taq* (TaKaRa Bio, Kusatsu, Japan) and analyzed in 96-well optical reaction plates, using the standard curve method. A. fumigatus 18S rRNA gene fragments were amplified with the primer pair 5′-GGCCCTTAAATAGCCCGGT-3′ and 5′-TGAGCCGATAGTCCCCCTAA-3′ and the hybridization probe 5′-FAM-AGCCAGCGGCCCGCAAATG-TAMRA-3′, where FAM is 6-carboxyfluorescein and TAMRA is 6-carboxytetramethylrhodamine. Each 25-μl reaction solution contained 50 ng of DNA from mouse lungs and 200 nM probe. The PCR protocol was as follows: incubation at 50°C for 2 min and 95°C for 10 min, followed by 55 cycles of 65°C for 1 min and 95°C for 15 s. The fluorescence was monitored at the end of each cycle to obtain a measure of the amount of PCR product formed. The cycle numbers at which each sample reached the threshold were determined, and the amount of A. fumigatus DNA in 50 ng of mouse lung DNA was evaluated from the standard curve with the cycle numbers and log_2_ concentrations of 0.05 to 50,000 pg of DNA from A. fumigatus. All animal studies were approved by the Ethics Review Committee for Animal Experimentation of Nihon University. A. fumigatus studies were approved by the Microbial Safety Management Committee of the Nihon University School of Pharmacy (E-H25-001).

### Statistical analysis.

Results are expressed as means ± standard errors of the means (SEM). For comparison between groups, either ordinary one-way analysis of variance (ANOVA) with Tukey's *post hoc* comparison or the Kruskal-Wallis ANOVA with Dunn's *post hoc* comparison test were performed. Statistical significance was defined as a *P* value of <0.05.

## Supplementary Material

Supplemental material
