# Supplementary material for: In Vitro and In Vivo Efficacy of a Novel and Long-Acting Fungicidal Azole, PC1244, on Aspergillus fumigatus Infection
Source: Antimicrob Agents Chemother. 2018 Apr 26;62(5):e01941-17. doi: 10.1128/AAC.01941-17 (PMC5923123; doi:10.1128/AAC.01941-17)
Supplement: Supplemental material [file supp_62_5_e01941-17__index.html]

Supplemental material 

# *In Vitro* and *In Vivo* Efficacy of a Novel and Long-Acting Fungicidal Azole, PC1244, on Aspergillus fumigatus Infection

## Supplemental material

- Supplemental file 1 -

  Supplemental Figure S1

  PDF, 50K
