# Supplementary material for: In Vitro and In Vivo Efficacy of a Novel and Long-Acting Fungicidal Azole, PC1244, on Aspergillus fumigatus Infection
Source: Antimicrob Agents Chemother. 2018 Apr 26;62(5):e01941-17. doi: 10.1128/AAC.01941-17 (PMC5923123; doi:10.1128/AAC.01941-17)
Supplement: Supplemental material [file AAC.01941-17_zac005187074s1.pdf]

Supplement FIGURE 1

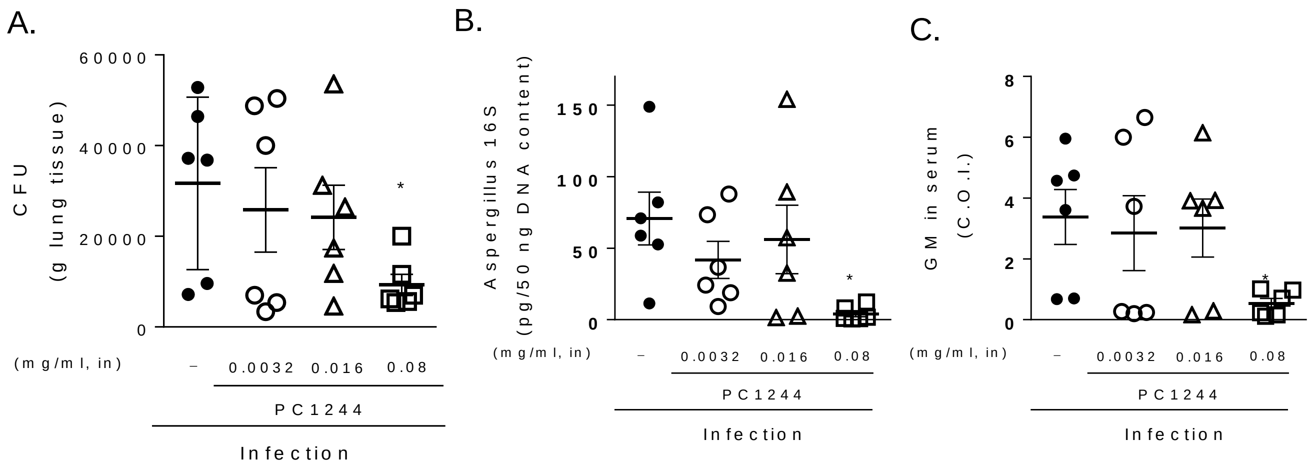

Supplement material FIG 1 *In vivo* pilot study to test antifungal activity of PC1244 against *A. fumigatus in vivo*. PC1244 were given intranasally on days 1, 2 and 3 post *A. fumigatus* inoculation, and fungal load (CFU) in lung (A), fungal load (PCR) in lung (B), galactomannan (GM) in serum (C) were evaluated in the lung or blood collected on day 3 post infection. (N=5~6). Each horizontal bar was presented as mean ± SD from 5~6 mice per group. * *P*<0.05.
